# CD5L deficiency attenuate acetaminophen-induced liver damage in mice via regulation of JNK and ERK signaling pathway

**DOI:** 10.1038/s41420-021-00742-3

**Published:** 2021-11-08

**Authors:** Mengjing Li, Tao Ling, Fengmeng Teng, Chao Hu, Zhongping Su, Chen Zhang, Xiang Li, Ting Zhao, Xianmin Mu, Yingchang Li, Jinshun Pan, Qiang You

**Affiliations:** 1 Department of Biotherapy, Department of Geriatrics, Second Affiliated Hospital of Nanjing Medical University, Nanjing, 210011 China; 2 Affilated Hospital of Nanjing University of Chinese Medicine, Nanjing, 210029 China; 3 Affiliated Cancer Hospital & Institute of Guangzhou Medical University, Guangzhou, 510095 China; 4 Key Laboratory of Cell Homeostasis and Cancer Research of Guangdong Higher Education Institutes, Guangzhou Medical University, Guangzhou, 510182 China

**Keywords:** Cell signalling, Immunology

## Abstract

CD5 molecule like (CD5L), a member of the scavenger receptor cysteine-rich domain superfamily, plays a critical role in immune homeostasis and inflammatory disease. Acetaminophen (APAP) is a safe and effective antipyretic analgesic. However, overdose may cause liver damage or even liver failure. APAP hepatotoxicity is characterized by extensive necrotic cell death and a sterile inflammatory response, in which the role of CD5L remains to be investigated. In this study, we found that the expression of CD5L was increased in the livers of mice after APAP overdose. Furthermore, CD5L deficiency reduced the increase of alanine transaminase (ALT) level, histopathologic lesion area, c-Jun N-terminal kinase (JNK)/extracellular signal-regulated kinase (ERK) phosphorylation level, Transferase-Mediated dUTP Nick End-Labeling positive (TUNEL^+^) cells proportion, vascular endothelial cell permeability and release of inflammatory cytokines induced by excess APAP. Therefore, our findings reveal that CD5L may be a potential therapeutic target for prevention and treatment of APAP-induced liver injury.

## Introduction

Acetaminophen (APAP), one of the most widely used analgesic-antipyretic in the United States, is safe at therapeutic doses. However, excess of APAP is capable for leading to a centrilobular hepatic necrosis [1]. N-acetyl-p-benzoquinoneimine (NAPQI), the toxic product of APAP metabolized by the cytochrome P450 system, is depleted by the hepatic reduced glutathione (GSH) antioxidant system. The unconsumed NAPQI leads to APAP protein adduct (APAP-AD) formation, which resulting oxidative stress of hepatocyte mitochondria that occurs in the first few hours [2]. Necrotic hepatocytes can activate the innate immune system to mediate irreversible secondary damage [3]. Various studies suggest that oxidative stress and proinflammatory factors such as interleukin-6 (IL-6) activate the JNK signaling pathway [4] and associated with ERK signaling pathway [5, 6].

CD5L, also termed as apoptosis inhibitor of macrophage (AIM), is a soluble protein mainly produced by macrophages and belongs to the scavenger receptor cysteine rich superfamily [7, 8]. Recent studies have shown that it can also be produced by other cells, such as Th17 cells [9], retinal epithelial cells [10] and lung epithelial cells [11]. Early studies suggested that CD5L can support macrophages survival [8]. Surprisingly, CD5L is found to be associated with various diseases such as lipid metabolic disease [12], hepatocellular carcinoma [13, 14], fungus induced peritonitis [15], acute kidney injury [16] and myocardial infarction [17] in later studies. Regarding the effect of CD5L in the liver disease, it has been proved that CD5L plasma levels are upregulated in patients with liver damage [18, 19]. In addition, CD5L is associated with the hepatic fibrosis [20] and hepatocellular carcinoma [14]. However, whether CD5L participates in the process of pathogenesis during APAP-induced liver injury has not been addressed to date.

In this study, results showed that the expression of CD5L was induced in the development of APAP-induced liver injury. Furthermore, CD5L-deficient reduced APAP-induced liver injury in mice by impairing the activation of JNK and ERK signaling pathways.

## Results

### CD5L deficiency attenuates APAP-induced liver injury in mice

**Fig. 1:**
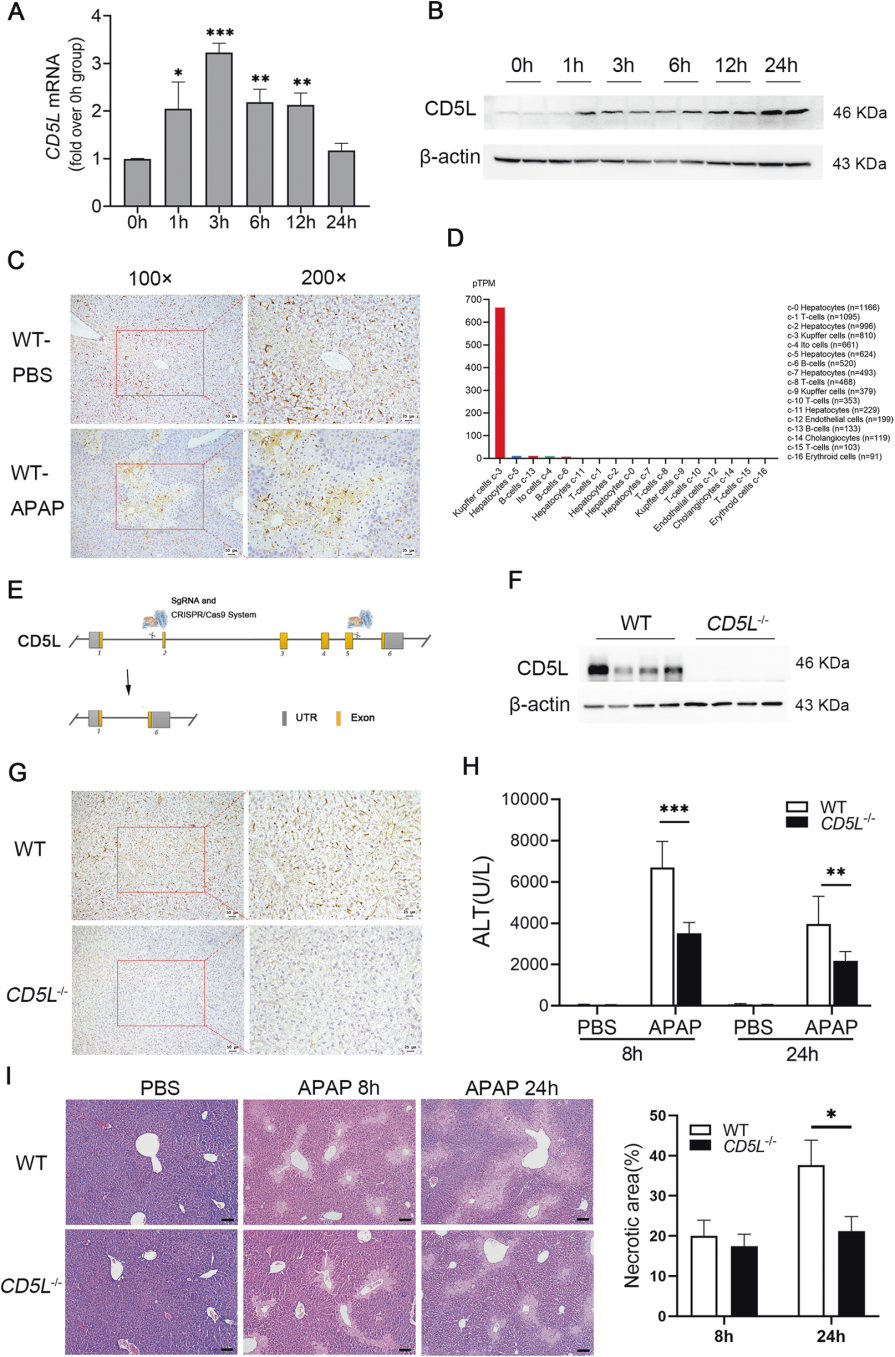
CD5L deficiency alleviates APAP-induced liver injury in mice. APAP was intraperitoneally injected into wild-type (WT) mice (*n* = 5 for each time point) at 300 mg/kg, and the CD5L level in the liver at specified time point was detected by **A** q-PCR and **B** western blotting; **C** Immunohistochemical staining of CD5L in liver sections of WT (*n* = 4 for each group) treated with PBS or APAP for 24 h, representative images are shown; **D** The expression of CD5L in human liver tissue (www.proteinatlas.org); **E** The diagram of CRISP/Cas9 CD5L knockout strategy; **F** The expression of CD5L protein in the liver of WT and CD5L^-/-^ mice was detected by western blotting; **G** Immunohistochemical staining of CD5L in liver sections of WT or *CD5L*^−/−^ mice (*n* = 4 for each group), representative images are shown; **H**, **I** Serum ALT and liver H&E staining at 8 h and 24 h after PBS or APAP injection in mice (*n* = 10 for each group). Necrotic area was measured by Image J. Representative images are shown. Scale: 100 μm. The results are presented as means ± SD of at least three independent experiments. *P < 0.05; **P < 0.01; ***P < 0.001.

To determine the role of CD5L in APAP-induced liver injury, male WT mice fasted overnight were treated with 300 mg/kg of APAP by intraperitoneal injection. CD5L mRNA level was increased two folds in livers after APAP treatment for 1 h, and the peak occurred at 3 h (Fig. 1A). Correspondingly, the CD5L protein level was significantly upregulated within 24 h in a time-dependent manner (Fig. 1B). Intriguingly, under physiological conditions, CD5L was only expressed in the interstitium of liver tissue, while APAP overdose induced its expression in liver parenchymal cells in the necrotic area around the central vein besides the mesenchymal cells (Fig. 1C). Correspondingly, human CD5L is expressed abundantly in Kuffer cells and slightly in hepatocytes according to the analysis from the website (www.proteinatlas.org) (Fig. 1D). These results suggested that CD5L is associated with APAP-induced liver injury.

To further determine the effect of CD5L on APAP-induced liver injury, CRISPR/cas9 gene editing technology was applied to construct CD5L gene knockout (*CD5L*^−/−^) mice successfully (Fig. 1E), in which CD5L expression proved to be absent in *CD5L*^−/−^ mice liver (Fig. 1F, G). After excessive APAP treatment, the elevation of serum hepatic injury index ALT in *CD5L*^−/−^ mice was much lower than that in WT mice (Fig. 1H) at 8 h and 24 h. Meanwhile, the lesion area caused by APAP in *CD5L*^−/−^ mice was much smaller than that in WT mice after treatment for 24 h, although no difference was observed at 8 h (Fig. 1I). Consequently, the results suggested that the absence of CD5L alleviated APAP-induced liver injury.

### CD5L deficiency reduces the activation of JNK and ERK signaling pathways

**Fig. 2:**
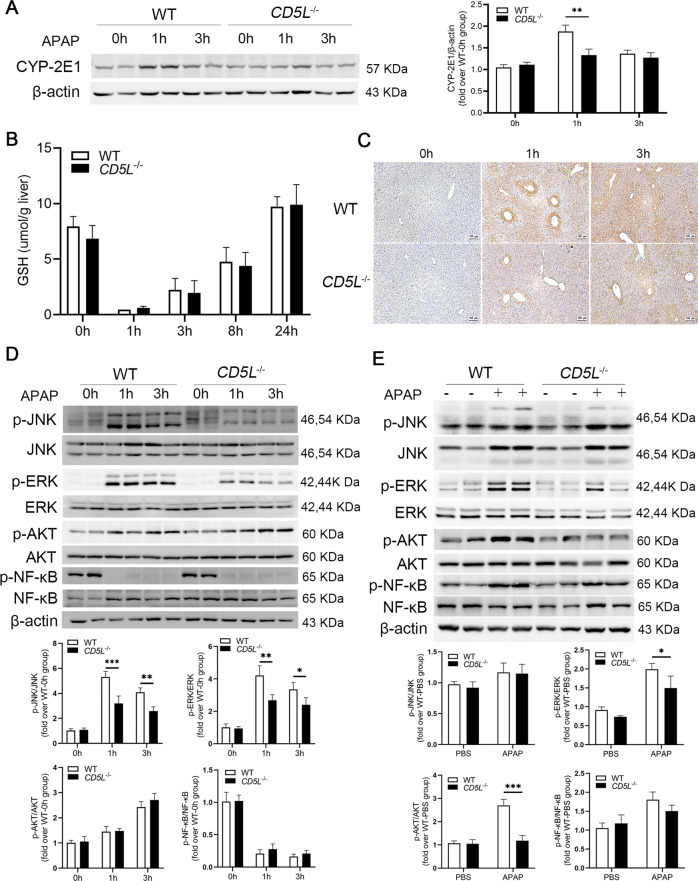
CD5L deficiency inhibits the activation of JNK and ERK signaling pathways. **A** CYP-2E1 protein in the liver of each group was examined by Western blotting at specified time point after administration. A representative blot and the means ± SD of three independent analyses were shown; **B**, **C** Level of GSH and APAP-AD in liver at specified time point after administration. The levels of specific proteins in WT and *CD5L*^−/−^ mouse liver were detected by western blotting after treatment with PBS or APAP for 0,1,3 h (**D**) and 24 h (**E**). The phosphorylated proteins were evaluated by western blotting analysis normalized to total proteins. A representative blot and the means ± SD of three independent analyses. *P < 0.05; **P < 0.01; ***P < 0.001.

Cytochrome P450 2E1 (CYP2E1) is recognized as the most important enzyme for initiation of APAP-induced toxicity since CYP2E1 knockout mice have a resistance to APAP-induced liver injury [21]. CYP2E1 protein showed a transient increase at 1 h in WT mice and had no change in *CD5L*^−/−^ mice (Fig. 2A). We next examined the changes of GSH and APAP-AD in liver tissue. Although we found no difference in glutathione between the two groups at different time points after APAP treatment (Fig. 2B), the APAP-AD was less in the liver tissue of *CD5L*^−/−^ mice after APAP overdose (Fig. 2C). The phosphorylation levels of JNK and ERK were dramatically lower in *CD5L*^−/−^ mice than those in WT mice at the first 3 h after APAP administration which is the critical timing for APAP hepatotoxicity, while no significant difference of p-AKT and p-NF-κB was observed (Fig. 2D). However, at 24 h after APAP treatment, *CD5L*^−/−^ mice exhibited weaker p-ERK, significantly lower p-AKT, and a trend to lower p-NF-κB expression compared to WT mice (Fig. 2E).

### CD5L protein activates JNK and ERK signaling pathway in mouse hepatocytes in vitro

**Fig. 3:**
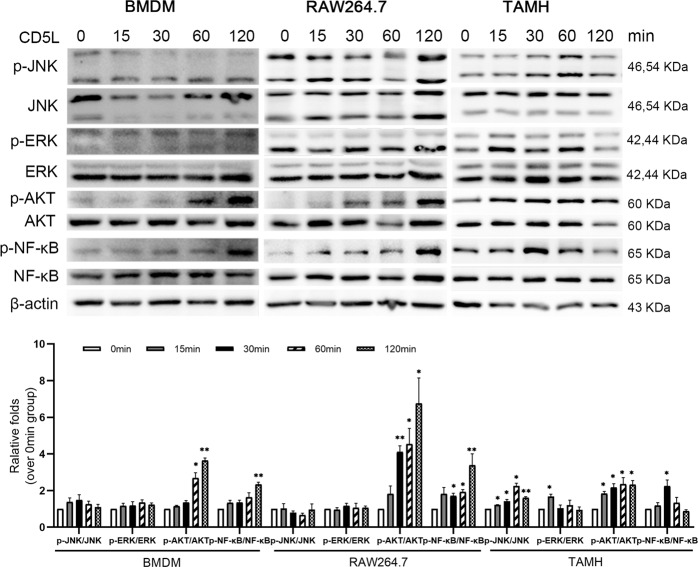
CD5L protein activates mouse macrophages and hepatocytes in vitro. CD5L protein (1 μg/ml) was co-incubate with BMDMs, RAW264.7 cells and TAMH cells, respectively. Cell proteins were collected at 0, 15, 30, 60, and 120 min, and the protein levels were detected by western blotting. The AKT, ERK, JNK, and NF-κB phosphorylation were evaluated using total ERK, NF-κB, AKT, and JNK as controls. The representative results from three independent experiments are shown and the data are presented as means ± SD. *P < 0.05; **P < 0.01.

To identify the role of CD5L protein in vitro, mouse bone marrow-derived macrophages (BMDMs) and macrophage cell line RAW264.7 cells were incubated with mouse CD5L protein (1 μg/ml), respectively. The phosphorylation of NF-κB and AKT were increased after the stimulation by CD5L in macrophages, while no change of p-JNK and p-ERK was observed (Fig. 3). To determine whether CD5L has effects on liver parenchymal cells other than through inflammatory cells, mouse hepatocytes (the transforming growth factor-α transgenic mouse hepatocyte, TAMH) were treated with recombinant CD5L protein directly. The phosphorylation levels of AKT, ERK, JNK, and NF-κB proteins in TAMH cells were all increased upon CD5L treatment (Fig. 3).

### CD5L deficiency decreases the expression of inflammatory factors in liver

**Fig. 4:**
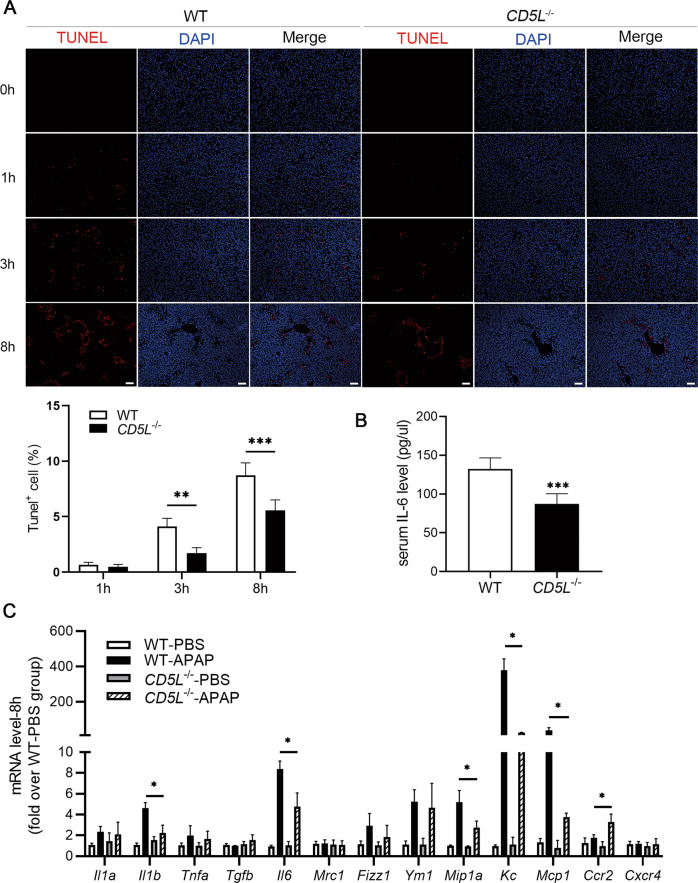
Lacking of CD5L reduces the level of inflammatory cytokines in the liver. **A** DNA fragmentation induced by APAP in mouse livers was detected by TUNEL assay at different tine point after APAP treatment. Scale: 100 μm. **B** The serum IL-6 concentration of mice in the two groups at 8 h after APAP treatment was measured by ELISA; **C** Q-PCR was used to detect mRNA levels of cytokines in the liver of WT and *CD5L*^−/−^ mice at 8 h after APAP treatment (*n* = 4 for each genotype at each time point). The results are presented as means ± SD of at least three independent experiments. *P < 0.05; **P < 0.01; ***P < 0.001.

The TUNEL assay was used to detect APAP-induced hepatocyte necrosis, and the results showed that the ratio of TUNEL^+^ cells in *CD5L*^−/−^ mice liver tissue was decreased markedly (Fig. 4A). To further explore the effect of CD5L on APAP-induced inflammation, the expression level of inflammatory cytokines in the livers of *CD5L*^−/−^ and WT mice was examined. The concentration of IL-6 was much less in the serum of APAP treated *CD5L*^−/−^ mice than that in WT mice (Fig. 4B). At 8 h after receiving APAP injection, the mRNA levels of inflammatory factor IL-1β, IL-6, MIP-1α, KC, and MCP-1 were decreased and CCR2 was increased in *CD5L*^−/−^ mice compared to those in WT mice (Fig. 4C). Therefore, a lack of CD5L leads to a reduction in APAP-induced hepatic inflammatory response.

### CD5L deficiency affects the infiltration of inflammatory cells

**Fig. 5:**
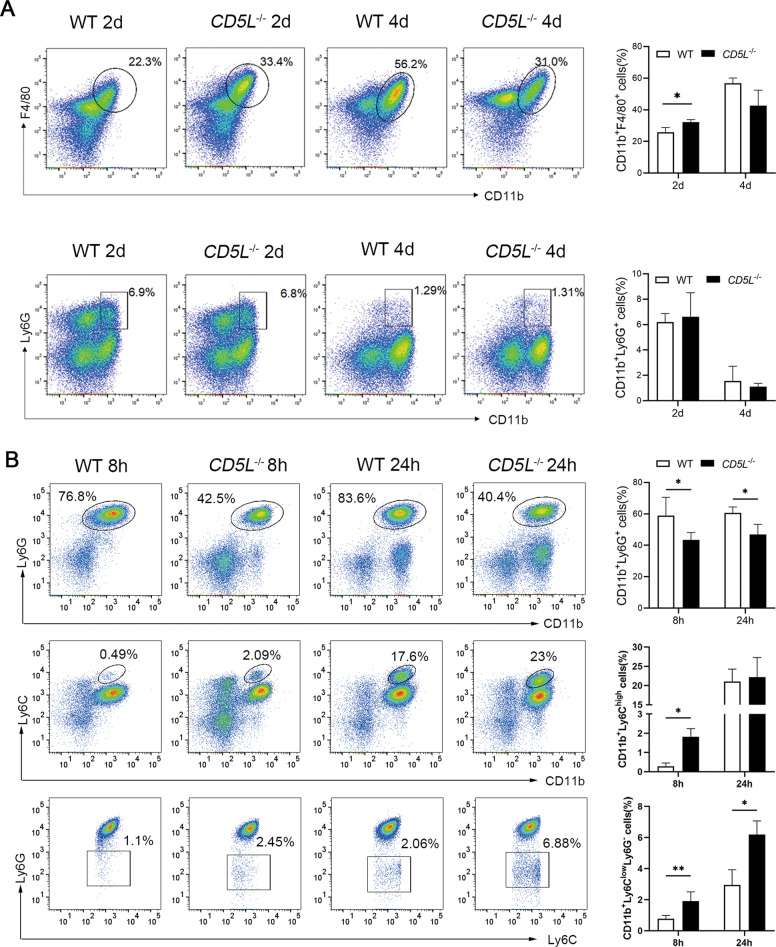
CD5L changes the infiltration of neutrophils and monocytes in mice. **A** Inflammatory cells isolated from mice at 2 and 4 days after intraperitoneal injection of 4% thioglycolate were analyzed by flow cytometry for macrophages (CD11b^+^F4/80^+^) and neutrophils (CD11b^+^Ly6G^+^) (*n* = 5 for each group at each time point); **B** Non parenchymal cells (NPCs) isolated from the liver of WT and *CD5L*^−/−^ mice after APAP treatment for 8 h and 24 h (*n* = 5 for each group at each time point) analyzed by flow cytometry. The expression of neutrophils (CD11b^+^Ly6G^+^), CD11b^+^Ly6C^high^ monocytes, and CD11b^+^Ly6C^low^ monocytes (from top to bottom) after APAP treatment for 8 h and 24 h. The results are presented as means ± SD of at least three independent experiments. *P < 0.05; **P < 0.01.

In order to further understand the effect of CD5L on the infiltration of inflammatory cells, thioglycolate was injected into the abdominal cavity of mice. After 2 days of treatment, there were more peritoneal macrophages in *CD5L*^−/−^ mice than that of WT mice. Interestingly, the amounts of macrophages in the *CD5L*^−/−^ mice were less than that of WT mice at 4 days post treatment although no statistical significance (Fig. 5A). In addition, there were more neutrophils after 2 days of treatment than that of 4 days, but no difference between the two groups (Fig. 5A). We concluded that CD5L knockout could affect the increase of infiltration of macrophages in the abdominal cavity.

Previous studies have shown that neutrophils and macrophages exert important roles in APAP-induced liver inflammation [22–24]. Flow cytometric analysis showed that the percentage of hepatic-infiltrating neutrophils (CD11b^+^Ly6G^+^) was obviously lower in the livers from *CD5L*^−/−^ mice after APAP injection than those in WT mice (Fig. 5B). Eight hours after APAP treatment, the percentage of CD11b^+^Ly6C^high^ (Ly6C^hi^) monocytes and CD11b^+^Ly6C^low^Ly6G^-^ (Ly6C^lo^) monocytes in the liver of *CD5L*^−/−^ mice were higher than those in WT mice. As for 24 h, the Ly6C^lo^ monocytes were much higher in *CD5L*^−/−^ mice, and there was no significant difference between the two groups in Ly6C^hi^ monocytes (Fig. 5B). These data demonstrate that CD5L deficiency can reduce the infiltration of neutrophils and improve the infiltration of monocytes in the APAP model.

### CD5L deficiency enhances hepatocytes proliferation

**Fig. 6:**
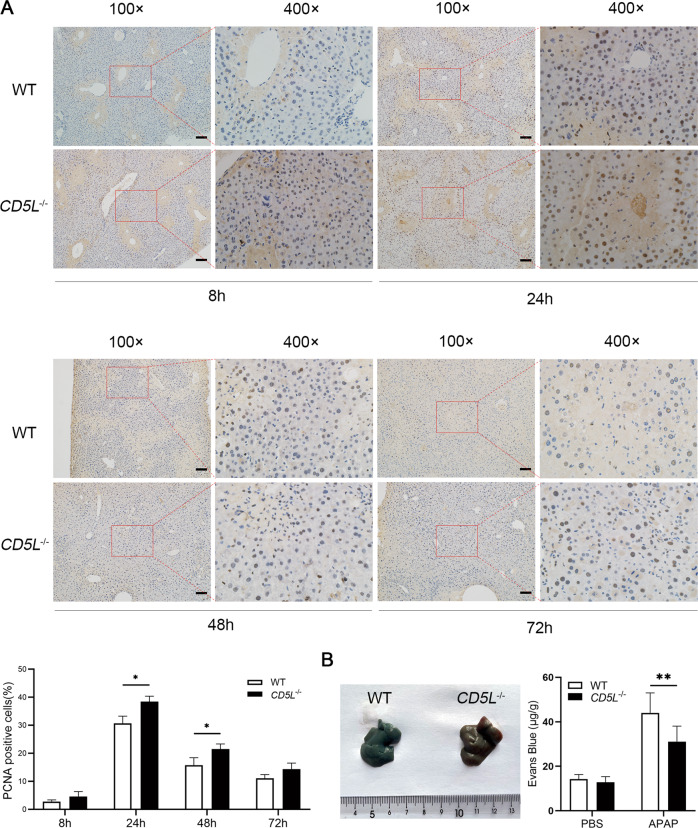
The proliferation of hepatocytes is increased in *CD5L*^−/−^ mice. **A** Immunohistochemical staining of PCNA in WT and *CD5L*^−/−^ mouse liver at the indicated time point after PBS or APAP treatment (*n* = 5 for each group at each time point). Deeply stained nucleus indicates positive results. The ratio of PCNA positive cells to total hepatocytes was calculated. Representative images are shown. Scale: 100 μm. **B** Vascular endothelial permeability after APAP treatment for 24 h was determined by Evans Blue assay (*n* = 8 for each group). The results are presented as means ± SD of at least three independent experiments. *P < .05; **P < 0.01.

It has been reported that neutrophils and macrophages contribute to the tissue repair process [25]. Therefore, the expression of PCNA was examined to observe the proliferation of liver cells. At 8 h after APAP treatment there was few positive staining in the injured liver tissues, while abundant positive nuclei were observed at 24 h. Intriguingly, the proportion of positive cells in *CD5L*^−/−^ mice was significantly higher than that in WT mice at 24 h and 48 h, although there was no difference at 72 h (Fig. 6A). This suggests an increase in proliferation of hepatocytes in APAP-induced liver injury following CD5L deficiency. Furthermore, the permeability of *CD5L*^−/−^ mice endothelial cells was weaker than that of WT after liver injury (Fig. 6B), indicating that *CD5L*^−/−^ mice had better ability in the repair of damage.

## Discussion

APAP-induced hepatotoxicity is confirmed as a ‘two-hit’ process: the oxidative stress of APAP metabolites and acute necrotic inflammatory response [22]. The immunomodulatory effect of CD5L is critical for the control of immune homeostasis [26]. In this study, increased expression of CD5L was observed in the liver of mice treated with excessive APAP. CYP2E1 is necessary to convert APAP into toxic products and the damage caused by APAP can be reduced after CYP2E1 knocked out [27]. There was a transient increase of CYP2E1 protein in WT mice after APAP treatment for 1 h, while not observed in *CD5L*^−/−^ mice. Meanwhile, consistent GSH depletion prompt that it made no difference in the detoxification ability of NAPQI between the two group mice. Attenuated expression of CYP2E1, resulting in the reduction of APAP-AD, was the cause of the reduced damage of *CD5L*^−/−^ mice.

APAP-AD induces the production of reactive oxygen species (ROS), which leads to continuous activation of JNK via several pathways. Then the activated JNK translocate to the mitochondrial membrane and further lead to hepatocyte death [4]. CD5L deficiency attenuated JNK activation resulting in the weaker liver damage. In addition, CD5L protein directly activates the JNK signaling pathway in hepatocytes but not in macrophages, which may play important role in promoting hepatocyte damage, exactly as abnormal CD5L accumulation can also lead to kidney and hepatocellular carcinoma cells injury [28]. Coinciding with the increased expression of p-JNK, CD5L also improved the level of p-ERK in hepatocytes. APAP-induced hepatocyte necrosis is accompanied by exposure of nuclear DNA fragments [29], therefore, fewer TUNEL^+^ signals detected in the livers of injured *CD5L*^−/−^ mice indicate reduced necrotic cells.

In the present study, our experiments demonstrate that CD5L protein can directly activate JNK and ERK signaling pathways in hepatocytes. The presence of CD5L may contribute to the promotion of mitochondrial oxidative stress by JNK in hepatocytes after APAP administration. CD5L deficiency reduces the toxic substance produced by APAP metabolism at an early stage, thereby attenuating the activation of JNK and ERK signaling pathways to alleviate APAP-induced injury. The detailed mechanism by which CD5L affects hepatocytes and macrophages in the APAP-induced liver injury requires further investigation.

Numerous studies have shown that infiltrating neutrophils and macrophages are involved in the process of acute liver inflammation in the mouse hepatitis model [30–33]. Upon APAP overdosage, neutrophils accumulate in the liver and mediate injury and inflammation [30, 34, 35]. When the infiltration of neutrophils is blocked, the hepatotoxicity can be significantly prevented. Chemokines (KC and MIP-1α), which account for neutrophils infiltration [36], were much lower in *CD5L*^−/−^ mice. In thioglycolate induced peritonitis, the acute inflammatory response peaks in 1–2 days and begins to subside in 3–4 days [37], and CD5L did not influence the neutrophil recruitment in our results. Therefore, the decreased neutrophils infiltration in the *CD5L*^−/−^ mouse liver might be due to the difference in activation of JNK signaling pathway caused by APAP in the early stage.

Our previous study indicates that both resident and infiltrating macrophages play important roles in liver blood vessel repair [38]. In aseptic peritonitis of mice, more macrophages were observed after 2 days and fewer after 4 days in *CD5L*^−/−^ mice than that in WT mice, prompting that the knockout of CD5L gene seemed to facilitate the recruitment of monocytes. It has been reported that the number of resident macrophages in the liver is rapidly reduced and bone marrow-derived monocytes infiltrate into the liver after acetaminophen poisoning [39]. The phenotype and function of monocytes depend on different microenvironments [40]. The activation pathways of monocytes are divided into classical and alternative activation. Classical activation into M1 macrophages (Ly6C^hi^) can release proinflammatory mediators to promote inflammation, while alternative activation into M2 macrophages (Ly6C^lo^) can release anti-inflammatory mediators that down-regulate inflammation and promote inflammatory repair [41]. Our results showed that Ly6C^lo^ monocytes were more abundant in *CD5L*^−/−^ mice. Combining with the results of PCNA assay, we concluded that CD5L deficiency can promote the enhancement of liver tissue repair ability by promoting the increase of Ly6C^lo^ monocytes.

As the hepatic vasculature represents a target of APAP-induced liver injury [42], Evans blue assay was performed to evaluate vascular permeability. CD5L deficiency relieved APAP-induced hepatic sinusoidal vascular endothelial cell permeability. These results indicated that the deletion of CD5L reduced the injury of endothelial cells, and it might also be the reason for the enhancement of repair.

In summary, our study identified that CD5L is involved in hepatotoxicity caused by APAP overdose and may be involved in injury repair. This discovery opened the door toward the research of acute hepatitis treatment, additional efforts might lead to the development of therapeutic targets for different stages of drug-induced hepatitis.

## Materials and methods

### Animals

Experiments were performed with 6–8 weeks old male C57BL/6J WT (Nanjing Biomedical Research Institute of Nanjing University). The mice were housed in a temperature-controlled environment with a 12 h light–dark cycle, and were allowed free access to water and food. All animal procedures were approved by the Laboratory Animal Core Facility of Nanjing Medical University.

### Construction of CD5L deficient mice

To achieve precise editing of specific gene sites in the mouse genome, we designed to knock out the *Cd5l* gene located in the C57BL/6J mouse genome Chr3, grcm38.p3 by using the CRISPR/Cas9 gene knockout technique. *Cd5l* gene consists of 6 exons. ATG initiation codon located in exon 1, and TGA termination codon located in exon 6. The sgRNA direct Cas9 endonuclease cleavage of *Cd5l* gene and create a DSB (double-strand break). Such breaks will be repaired, and result in the deletion of exon2–5. The sgRNA sequence (5’ to 3’) and protospacer adjacent motif (PAM) used are as follows, S1 (GGAAGGCACGAAGCCTCCAA, GGG); S2 (CTAGCCTCAAAGAACACCAT, GGG); S3 (AGAGCAGGTAAAGACGCCAC, TGG); S4 (CCTTGAGATTTGTACAGAGC, AGG). CD5L KO mice were generated by Nanjing Biomedical Research Institute of Nanjing University.

### In vivo induction of liver injury

The mouse model of APAP-induced liver injury was performed as previously described [38]. Simply, 6–8 weeks old male WT or *CD5L*^*−/−*^ mice were fasted for ~16 h (overnight) by removing food and replacing bedding to deplete glutathione levels, prior to intraperitoneal injection of PBS or APAP (300 mg/kg, Sigma-Aldrich, St. Louis, MO, USA) dissolved in heated phosphate buffer saline (PBS, 60 °C). Mice were randomly assigned to PBS group or APAP group.

### Cell culture and treatment

The transforming growth factor-α transgenic mouse hepatocyte (TAMH cells, a generous gift of Dr. Cynthia Ju, UTHealth, Houston, Texas) and RAW264.7 cells were cultured in DMEM/F12 or DMEM medium supplemented with 10% fetal bovine serum (FBS), 100 units/ml penicillin, and 100 μg/ml streptomycin. Cell lines were authenticated by Genetic Testing Biotechnology Corporation (Suzhou, China) using short tandem repeat markers profiling, and tested negative for mycoplasma. Cells were cultured at 37 °C in a humidified 95% air, 5% CO_2_ atmosphere. The cells were starved for four hours in serum-free medium before treatment, and stimulated with 1 μg/ml of CD5L protein for specified duration to collect protein at each time point.

### Serum aspartate aminotransferase (AST) and alanine aminotransferase (ALT) analysis

Blood was collected from the post-orbitalvenous plexus at 8 and 24 h after APAP injection. The serum was separated by centrifugation and the levels of AST and ALT in the serum were measured with an automated chemical analyzer (MODULAR EVO 4200, Switzerland).

### Immunohistochemical assays

The isolated liver tissues from the treated mice were fixed in 10% formalin for 24 h followed by processing and paraffin embedding. Sections (4μm) of paraffin-embedded tissues were stained with hematoxylin and eosin (H&E). Immunohistochemical (IHC) staining was performed using CD5L antibody (#50020-T24, Sino Biological, Beijing, China), PCNA antibody (#13110, Cell Signaling Technology, Beverly, MA, USA) and sheep anti-APAP polyclonal antibody (#0016-0104, Bio-Rad, Düsseldorf, Germany). Tissue sections stained with specific antibodies were evaluated by light-microscopic (Olympus IX51, Japan). Pathological images of tissue sections were analyzed by Image J (1.51j8).

### Western blotting

Total proteins were prepared from mouse livers or cultured cell samples using STE buffer or RIPA lysis buffer containing protease and phosphatase inhibitors cocktail (Roche, Basel, Switzerland). After protein quantification with Pierce™ Rapid Gold BCA Protein Assay Kit (Thermo Fisher Scientific, Waltham, MA, USA), the lysate supernatants were heated in sodium dodecyl sulfate-polyacrylamide gelelectrophoresis (SDS-PAGE) sample-loading buffer. Protein extracts were separated on 8–15% SDS-polyacrylamide gels and transferred to the PVDF membrane. After blocking in 5% bovine serum albumin (BSA), the membrane was probed with specific primary antibodies followed by horseradish peroxidase conjugated antibody. The antibodies were used as follows: Phospho-ERK (#4370), total ERK (#4695), Phospho-AKT (#9271), total AKT (#4691), Phospho-JNK (#9251), total JNK (#9258), Phospho-NF-κB P65 (#3033), total NF-κB P65 (#8242). All above antibodies are from Cell Signaling Technology (Beverly, MA, USA). Anti-CD5L antibody (ab45408) is from Abcam (Cambridge, UK). Antibodies are diluted in the proportion indicated in the instructions.

### Hepatic reduced glutathione (GSH) content measurement

Hepatic GSH was measured using a Glutathione Assay Kit (Sigma-Aldrich, St. Louis, MO, USA) as followed by the manufacturer’s protocol.

### Transferase-mediated dUTP nick end-labeling (TUNEL) assay

To measure the hepatic nuclear DNA strand breaks, paraffin sections were stained with TUNEL method using an In Situ Cell Death Detection Kit, TMR red (Roche, Basel, Switzerland) according to the manufacturer’s protocols.

### Permeability assay using Evans blue dye

Mice were injected intraperitoneally with Evans blue dye (Sigma-Aldrich, St. Louis, MO, USA) at a dose of 20 mg/kg 24 h after APAP treatment. Four hours after administration, mouse liver tissues were perfused in situ with Hanks’ Balanced Salt Solution (HBSS) to remove excess dye remaining in the circulation. Livers were excised and placed in formamide (4 mL/g tissue) and incubated at 45 °C for 16 h to allow dye extraction. The supernatant was measured using spectrophotometry at the wavelength of 630 nm. The amount of Evans blue dye in the tissue was calculated from the standard curve of known Evans blue concentrations.

### Quantitative real-time PCR for mRNA expression analyses

Total RNA was extracted from liver tissues collected at 8 h and 24 h after APAP injection using the Ultrapure RNA kit (Thermo Fisher Scientific, Invitrogen, MA, USA) and transcribed into cDNA using the reverse transcription kit (Thermo Fisher Scientific, Waltham, MA, USA). Subsequently, the resultant cDNA was amplified with the Maxima SYBR-Green/Rox q-PCR Master Mix 2X kit (Thermo Fisher Scientific, Waltham, MA, USA) using the Step One Plus Real-Time PCR System (Thermo Fisher Scientific, Waltham, MA, USA). Primers used in the PCR-reactions were synthesized in Invitrogen (Shanghai, China) as followed: GAPDH (CAT CAC TGC CAC CCA GAA GAC TG, ATG CCA GTG AGC TTC CCG TTC AG); IL-1α (ACG GCT GAG TTT CAG TGA GAC C, CAC TCT GGT AGG TGT AAG GTG C); IL-1β (TGG ACC TTC CAG GAT GAG GAC A, GTT CAT CTC GGA GCC TGT AGT G); TNF-α (GGT GCC TAT GTC TCA GCC TCT T, GCC ATA GAA CTG ATG AGA GGG AG); TGF-β (TGA TAC GCC TGA GTG GCT GTC T, CAC AAG AGC AGT GAG CGC TGA A); IL-6 (TAC CAC TTC ACA AGT CGG AGG C, CTG CAA GTG CAT CAT CGT TGT TC); Mrc1 (GCT TCC GTC ACC CTG TAT GC, TCA TCC GTG GTT CCA TAG ACC); Fizz1 (CCA ATC CAG CTA ACT ATC CCT CC, ACC CAG TAG CAG TCA TCC CA); YM1 (CAG GTC TGG CAA TTC TTC TGA A, GTC TTG CTC ATG TGT GTA AGT GA); MIP-1α (TGT ACC ATG ACA CTC TGC AAC, CAA CGA TGA ATT GGC GTG GAA); KC (ACT GCA CCC AAA CCG AAG TC, TGG GGA CAC CTT TTA GCA TCT T); MCP1 (TGT ACC ATG ACA CTC TGC AAC, CAA CGA TGA ATT GGC GTG GAA); CCR2 (ATC CAC GGC ATA CTA TCA ACA TC, TCG TAG TCA TAC GGT GTG GTG); CXCR4 (GAC TGG CAT AGT CGG CAA TG, AGA AGG GGA GTG TGA TGA CAA A). The relative gene expression levels were calculated using the comparative 2^−ΔΔCt^ method.

### Enzyme-linked immunosorbent assay (ELISA)

The concentration of IL-6 in the serum of mice was detected by ELISA kit (Bio Legend, San Diego, CA, USA) as followed by the manufacturer’s protocol.

### Isolation of bone marrow-derived macrophages (BMDM)

The donor mice were sacrificed and leg bones were collected. The bone marrow was washed out with RPMI medium, and cell suspensions were filtered through a 70 µm cell strainer (BD Falcon, Bedford, MA, USA). The red blood cells were lysed and removed after centrifugation. Then, the cells were incubated in petri dish for three days with RPMI medium containing 10% FBS and 10 ng/ml macrophage colony-stimulating factor (M-CSF) at 37 °C in a humidified 95% air, 5% CO_2_ atmosphere. Each dish was supplemented with 4 ml of RPMI medium containing 10% serum and M-CSF (14 ng/ml) and cultured for three additional days. Then these cells were subjected to subsequent protein-related stimulation tests.

### Isolation of liver non-parenchymal cells (NPCs)

LNPCs isolation was following a previously established method [33]. In brief, after perfusion with HBSS containing ethylenebis (oxyethylenenitrilo) tetraacetic acid (EGTA), the liver was excised and homogenized with HBSS containing 0.5% FBS. The tissue was passed through a 100μm cell strainer (BD Falcon, Bedford, MA, USA). Then, 30% percoll (Sigma-Aldrich, St. Louis, MO, USA) was used to isolate liver NPCs. Red blood cells were further lysed with red blood cell lysing buffer. Finally, these cells were resuspended in HBSS containing 2% FBS for antibody staining.

### Isolation of peritoneal immunocytes

Thioglycolate (BD Falcon, Bedford, MA, USA) was dissolved in ddH_2_O at a rate of 4%, then cooled to 4 °C temperature after autoclaved sterilization at 121 °C for 20 min, and each mouse was intraperitoneally injected with 3 ml thioglycolate solution. Two or 4 days later, the mice were intraperitoneally washed with 10 ml of cold PBS. Infiltrated inflammatory cells were collected from the peritoneal lavage fluids by centrifugation.

### Flow cytometry

Freshly isolated LNPCs were incubated with mouse Fc receptor blocker to prevent non-specific binding. Then, the cells were incubated with various staining antibodies, including APC Cyanine7conjugated anti-mouse CD45 (clone 30F11, #130-105-506; Miltenyi Research Inc. San Diego, CA), PE-vio770 conjugate anti-mouse CD11b (clone M1/70, #25-0112-82, eBioscience), APC conjugated anti-mouse F4/80 (clone BM8, #17-4801-82, eBioscience), FITC-conjugated anti-mouse Ly6C (clone AL21, #553104, BD Biosciences, San Jose, CA) or PE-conjugated anti-mouse Ly6G (clone RB6-8C5, #561084, BD Biosciences).

### Quantification and statistical analysis

All data were analyzed using GraphPad Prism software (version 8.0.2). Data are expressed as mean ± SD. Comparisons between experimental groups were conducted using ANOVA. For all experiments similar variances between groups were observed. Normal distribution of samples was determined. Differences were considered significant when *p* < 0.05. All the experiments were repeated at least three times.

## Data Availability

The data that support the findings of this study are available from the corresponding author upon reasonable request.

## References

[1] Larson AM, Polson J, Fontana RJ, Davern TJ, Lalani E, Hynan LS (2005). Acetaminophen-induced acute liver failure: results of a United States multicenter, prospective study. Hepatology.

[2] Bunchorntavakul C, Reddy KR (2013). Acetaminophen-related hepatotoxicity. Clin Liver Dis..

[3] Maher JJ (2009). DAMPs ramp up drug toxicity. J Clin Invest.

[4] Lee DH, Jung YS, Yun J, Han SB, Roh YS, Song MJ (2020). Peroxiredoxin 6 mediates acetaminophen-induced hepatocyte death through JNK activation. Redox Biol..

[5] Widjaja AA, Dong J, Adami E, Viswanathan S, Ng B, Pakkiri LS (2021). Redefining IL11 as a regeneration-limiting hepatotoxin and therapeutic target in acetaminophen-induced liver injury. Sci Transl Med.

[6] Zhang J, Zhang S, Bi J, Gu J, Deng Y, Liu C (2017). Astaxanthin pretreatment attenuates acetaminophen-induced liver injury in mice. Int Immunopharmacol..

[7] Gebe JA, Kiener PA, Ring HZ, Li X, Francke U, Aruffo A (1997). Molecular cloning, mapping to human chromosome 1 q21-q23, and cell binding characteristics of Spalpha, a new member of the scavenger receptor cysteine-rich (SRCR) family of proteins. J. Biol. Chem..

[8] Miyazaki T, Hirokami Y, Matsuhashi N, Takatsuka H, Naito M (1999). Increased susceptibility of thymocytes to apoptosis in mice lacking AIM, a novel murine macrophage-derived soluble factor belonging to the scavenger receptor cysteine-rich domain superfamily. J Exp Med.

[9] Wang C, Yosef N, Gaublomme J, Wu C, Lee Y, Clish CB (2015). CD5L/AIM regulates lipid biosynthesis and restrains Th17 cell pathogenicity. Cell.

[10] Iannaccone A, Hollingsworth TJ, Koirala D, New DD, Lenchik NI, Beranova-Giorgianni S (2017). Retinal pigment epithelium and microglia express the CD5 antigen-like protein, a novel autoantigen in age-related macular degeneration. Exp Eye Res.

[11] Li Y, Qu P, Wu L, Li B, Du H, Yan C (2011). Api6/AIM/Spalpha/CD5L overexpression in alveolar type II epithelial cells induces spontaneous lung adenocarcinoma. Cancer Res.

[12] Kurokawa J, Arai S, Nakashima K, Nagano H, Nishijima A, Miyata K (2010). Macrophage-derived AIM is endocytosed into adipocytes and decreases lipid droplets via inhibition of fatty acid synthase activity. Cell Metab..

[13] Maehara N, Arai S, Mori M, Iwamura Y, Kurokawa J, Kai T (2014). Circulating AIM prevents hepatocellular carcinoma through complement activation. Cell Rep..

[14] Aran G, Sanjurjo L, Barcena C, Simon-Coma M, Tellez E, Vazquez-Vitali M (2018). CD5L is upregulated in hepatocellular carcinoma and promotes liver cancer cell proliferation and antiapoptotic responses by binding to HSPA5 (GRP78). FASEB J..

[15] Tomita T, Arai S, Kitada K, Mizuno M, Suzuki Y, Sakata F (2017). Apoptosis inhibitor of macrophage ameliorates fungus-induced peritoneal injury model in mice. Sci Rep..

[16] Arai S, Kitada K, Yamazaki T, Takai R, Zhang X, Tsugawa Y (2016). Apoptosis inhibitor of macrophage protein enhances intraluminal debris clearance and ameliorates acute kidney injury in mice. Nat Med..

[17] Nishikido T, Oyama J, Shiraki A, Komoda H, Node K (2016). Deletion of Apoptosis Inhibitor of Macrophage (AIM)/CD5L Attenuates the Inflammatory Response and Infarct Size in Acute Myocardial Infarction. J Am Heart Assoc..

[18] Gangadharan B, Antrobus R, Dwek RA, Zitzmann N (2007). Novel serum biomarker candidates for liver fibrosis in hepatitis C patients. Clin Chem..

[19] Gray J, Chattopadhyay D, Beale GS, Patman GL, Miele L, King BP (2009). A proteomic strategy to identify novel serum biomarkers for liver cirrhosis and hepatocellular cancer in individuals with fatty liver disease. BMC Cancer.

[20] Barcena C, Aran G, Perea L, Sanjurjo L, Tellez E, Oncins A (2019). CD5L is a pleiotropic player in liver fibrosis controlling damage, fibrosis and immune cell content. EBioMedicine.

[21] Cheung C, Yu AM, Ward JM, Krausz KW, Akiyama TE, Feigenbaum L (2005). The cyp2e1-humanized transgenic mouse: role of cyp2e1 in acetaminophen hepatotoxicity. Drug Metab. Dispos..

[22] Zhang C, Feng J, Du J, Zhuo Z, Yang S, Zhang W (2018). Macrophage-derived IL-1alpha promotes sterile inflammation in a mouse model of acetaminophen hepatotoxicity. Cell Mol Immunol..

[23] Michael SL, Pumford NR, Mayeux PR, Niesman MR, Hinson JA (1999). Pretreatment of mice with macrophage inactivators decreases acetaminophen hepatotoxicity and the formation of reactive oxygen and nitrogen species. Hepatology.

[24] Antoniades CG, Quaglia A, Taams LS, Mitry RR, Hussain M, Abeles R (2012). Source and characterization of hepatic macrophages in acetaminophen-induced acute liver failure in humans. Hepatology.

[25] Bouchery T, Harris N (2019). Neutrophil-macrophage cooperation and its impact on tissue repair. Immunol Cell Biol..

[26] Gao X, Yan X, Zhang Q, Yin Y, Cao J (2019). CD5L contributes to the pathogenesis of methicillin-resistant Staphylococcus aureus-induced pneumonia. Int Immunopharmacol..

[27] Shayiq RM, Roberts DW, Rothstein K, Snawder JE, Benson W, Ma X (1999). Repeat exposure to incremental doses of acetaminophen provides protection against acetaminophen-induced lethality in mice: an explanation for high acetaminophen dosage in humans without hepatic injury. Hepatology.

[28] Takahata A, Arai S, Hiramoto E, Kitada K, Kato R, Makita Y (2020). Crucial Role of AIM/CD5L in the development of glomerular inflammation in IgA nephropathy. J Am Soc Nephrol..

[29] McGill MR, Sharpe MR, Williams CD, Taha M, Curry SC, Jaeschke H (2012). The mechanism underlying acetaminophen-induced hepatotoxicity in humans and mice involves mitochondrial damage and nuclear DNA fragmentation. J Clin Invest.

[30] He Y, Feng D, Li M, Gao Y, Ramirez T, Cao H (2017). Hepatic mitochondrial DNA/Toll-like receptor 9/MicroRNA-223 forms a negative feedback loop to limit neutrophil overactivation and acetaminophen hepatotoxicity in mice. Hepatology.

[31] Si Y, Tsou CL, Croft K, Charo IF (2010). CCR2 mediates hematopoietic stem and progenitor cell trafficking to sites of inflammation in mice. J. Clin. Invest.

[32] Laskin DL (2009). Macrophages and inflammatory mediators in chemical toxicity: a battle of forces. Chem Res Toxicol..

[33] You Q, Cheng L, Reilly TP, Wegmann D, Ju C (2006). Role of neutrophils in a mouse model of halothane-induced liver injury. Hepatology.

[34] Marques PE, Amaral SS, Pires DA, Nogueira LL, Soriani FM, Lima BH (2012). Chemokines and mitochondrial products activate neutrophils to amplify organ injury during mouse acute liver failure. Hepatology.

[35] Liu ZX, Han D, Gunawan B, Kaplowitz N (2006). Neutrophil depletion protects against murine acetaminophen hepatotoxicity. Hepatology.

[36] Knudsen E, Iversen PO, Van Rooijen N, Benestad HB (2002). Macrophage-dependent regulation of neutrophil mobilization and chemotaxis during development of sterile peritonitis in the rat. Eur J Haematol..

[37] Phillips BE, Geletzke AK, Smith PB, Podany AB, Chacon A, Kelleher SL (2016). Impaired recovery from peritoneal inflammation in a mouse model of mild dietary zinc restriction. Mol Nutr Food Res.

[38] You Q, Holt M, Yin H, Li G, Hu CJ, Ju C (2013). Role of hepatic resident and infiltrating macrophages in liver repair after acute injury. Biochemical Pharmacol..

[39] Gardner CR, Hankey P, Mishin V, Francis M, Yu S, Laskin JD (2012). Regulation of alternative macrophage activation in the liver following acetaminophen intoxication by stem cell-derived tyrosine kinase. Toxicol. Appl Pharm..

[40] Dong X, Liu J, Xu Y, Cao H (2019). Role of macrophages in experimental liver injury and repair in mice. Exp Ther Med.

[41] Shapouri-Moghaddam A, Mohammadian S, Vazini H, Taghadosi M, Esmaeili SA, Mardani F (2018). Macrophage plasticity, polarization, and function in health and disease. J Cell Physiol..

[42] Holt MP, Yin H, Ju C (2010). Exacerbation of acetaminophen-induced disturbances of liver sinusoidal endothelial cells in the absence of Kupffer cells in mice. Toxicol Lett..

